# Collective properties of evolving molecular quasispecies

**DOI:** 10.1186/1471-2148-7-110

**Published:** 2007-07-09

**Authors:** Michael Stich, Carlos Briones, Susanna C Manrubia

**Affiliations:** 1 Centro de Astrobiología (INTA-CSIC), Instituto Nacional de Técnica Aeroespacial, Ctra de Ajalvir, km 2, 28850 Torrejón de Ardoz (Madrid), Spain

## Abstract

**Background:**

RNA molecules, through their dual appearance as sequence and structure, represent a suitable model to study evolutionary properties of quasispecies. The essential ingredient in this model is the differentiation between genotype (molecular sequences which are affected by mutation) and phenotype (molecular structure, affected by selection). This framework allows a quantitative analysis of organizational properties of quasispecies as they adapt to different environments, such as their robustness, the effect of the degeneration of the sequence space, or the adaptation under different mutation rates and the error threshold associated.

**Results:**

We describe and analyze the structural properties of molecular quasispecies adapting to different environments both during the transient time before adaptation takes place and in the asymptotic state, once optimization has occurred. We observe a minimum in the adaptation time at values of the mutation rate relatively far from the phenotypic error threshold. Through the definition of a consensus structure, it is shown that the quasispecies retains relevant structural information in a distributed fashion even above the error threshold. This structural robustness depends on the precise shape of the secondary structure used as target of selection. Experimental results available for natural RNA populations are in qualitative agreement with our observations.

**Conclusion:**

Adaptation time of molecular quasispecies to a given environment is optimized at values of the mutation rate well below the phenotypic error threshold. The optimal value results from a trade-off between diversity generation and fixation of advantageous mutants. The critical value of the mutation rate is a function not only of the sequence length, but also of the specific properties of the environment, in this case the selection pressure and the shape of the secondary structure used as target phenotype. Certain functional motifs of RNA secondary structure that withstand high mutation rates (as the ubiquitous hairpin motif) might appear early in evolution and be actually frozen evolutionary accidents.

## Background

Molecular quasispecies are minimal evolutionary units able to evolve coherently and to adapt to environmental changes sufficiently fast to ensure survival. First introduced by Eigen [1], they represent a simple model of replicating molecular populations subject to high mutation rates and selective pressure. In the context of the origin of life, these populations may be an example of darwinian evolution prior to the appearance of organized cellular life [2]. The dynamics and fate of such populations in a given environment depend on several parameters, as the length of the molecules, the population size, or the mutation rate at replication. Too long molecules, too small populations, or too high mutation rates constitute main obstacles preventing the appearance and fixation of biological function.

Knowledge of the organizational properties of molecular quasispecies is essential to understand how novelty arises and is selected in populations of replicators. A minimal model to perform computational studies of molecular evolution consists of ensembles of replicating RNA sequences that fold into a secondary structure [3-5], which then becomes the target of selection. This simple separation of genotype and phenotype is crucial to take into account the degeneration between description levels (from sequence to structure and eventually function) [6,7] and to observe several different collective properties with relevant roles in evolution and adaptation, among them robustness against mutations [8] and structural diversity [9].

RNA secondary structure has been often used as a model of molecular phenotype. Its use has permitted to advance in the understanding of phenomena such as the discrete nature of evolutionary transitions [10], the influence of sequence degeneration in the adaptive properties of quasispecies [11], or the relation between genotypic and phenotypic error thresholds [12], to cite a few. Eventually, these advances aid in the interpretation of experimental observations with more complex populations, such as RNA viruses [13].

It is common knowledge that the high mutation rates under which RNA viral replication occurs maintain a large degree of diversity in their populations [14]. Due to their characteristics, they were proposed as the first natural case of quasispecies. Since then, an effort has been made to relate concepts arising from the theoretical definition of molecular quasispecies to empirical measures performed on actual natural populations. As a result, it has been proposed that RNA viruses evolve close to the error threshold [15] and that drift in the consensus sequence is not necessarily accompanied by changes in viability, due to degeneration [16]. The advantage of evolving robustness, a central property of quasispecies, has been recently demonstrated with subviral pathogens [17].

In this contribution, we analyze the structural and collective properties of an ensemble of RNA sequences subjected to replication at various mutation rates and subsequent selection depending on a target structure. Our aim is to understand how adaptation takes place in those populations, and the typical times required to find and fix a given secondary structure in the molecular ensemble. We observe that the population undergoes a kind of collective search and that some properties are delocalized (i.e. they are not found on any single molecule, but it is the average over the population that maintains them). We introduce the concept of consensus structure and demonstrate that, even at values of the mutation rate that forbid the fixation of a goal function in a significant fraction of molecules, the population as a whole reflects the selective pressure to which it is subjected. Finally, we suggest that quasispecies do not evolve too close to the error threshold, but have selected intermediate values of the mutation rate in order to optimize their response time to environmental changes. Thus, it is not the plain generation of diversity what is maximized through evolution, but the response time to external changes, which, in our case, is a combination of the time needed to find a given secondary structure (shorter at high mutation rates) and the time required to fix it in the population (shorter at low mutation rates).

## Results and Discussion

### Evolutionary algorithm

#### Selection and mutation

Our system model consists of a population of *N *replicating RNA sequences, each of length *n *nucleotides (nt). Population sizes and sequence length are kept constant during simulations. At the beginning of the simulation, every molecule of the population is initialized with a random sequence. As a molecule replicates, each nucleotide has a probability *μ *to be randomly replaced by another (or the same) type of nucleotide.

At each generation, the sequences are folded into secondary structures with help of the Vienna RNA package (see Methods). We define a target secondary structure which represents in a simple way optimal performance in the given environment. The secondary structure of molecules in the population is compared to the target structure, and the closer a secondary structure is to the target structure, the higher the probability *p*(*d*_*i*_) that the corresponding sequence *i *replicates. This probability is given by

*p*(*d*_*i*_) = *Z*^-1^exp(-*βd*_*i*_/*d*)

where *d*_*i *_is the distance between the structure corresponding to sequence *i *and the target structure, *d *is the average distance of the population to the target structure, *d *= ∑_*i*_*d*_*i*_/*N*, and Z=∑i=1Nexp⁡(−βdi/d)
 MathType@MTEF@5@5@+=feaafiart1ev1aaatCvAUfKttLearuWrP9MDH5MBPbIqV92AaeXatLxBI9gBaebbnrfifHhDYfgasaacH8akY=wiFfYdH8Gipec8Eeeu0xXdbba9frFj0=OqFfea0dXdd9vqai=hGuQ8kuc9pgc9s8qqaq=dirpe0xb9q8qiLsFr0=vr0=vr0dc8meaabaqaciaacaGaaeqabaqabeGadaaakeaacqWGAbGwcqGH9aqpdaaeWaqaaiGbcwgaLjabcIha4jabcchaWnaabmaabaGaeyOeI0ccciGae8NSdiMaemizaq2aaSbaaSqaaiabdMgaPbqabaGccqGGVaWlcqWGKbazaiaawIcacaGLPaaaaSqaaiabdMgaPjabg2da9iabigdaXaqaaiabd6eaobqdcqGHris5aaaa@42D2@. The parameter *β *denotes the selection pressure and takes the value *β *= 1 for all numerical results shown in this study. Generations in our simulation are non-overlapping and the offspring generation is calculated according to Wright-Fisher sampling at each time step, with fitness given by Eq. (1).

Starting with a population constituted by randomly chosen sequences, the population evolves through a transient regime until it reaches a statistically stationary state characterizing the asymptotic regime. Obviously, these processes depend on the parameters of the system, in particular on the mutation rate *μ*, as will be discussed in detail below.

#### Definitions

Secondary structures are denoted in standard bracket notation, where unpaired nucleotides are given by ".", and paired ones by "(" (upstream) and ")" (downstream). Matching brackets stand for paired bases. The target structures considered in this study correspond to biologically relevant RNA structures of 35 to 76 nt in length, and are listed in Table 1.

**Table 1:** Target Structures. Target structures used in the simulations.

Name	*n*	Structure in bracket notation
hairpin	35	..(((((((....(((...)))......)))))))
hammerhead	35	(((..((((....))))...((((...)))).)))
3-stem-loop	46	(((.((((....)))).((((....)))).((((....)))).)))
model tRNA	76	(((((((..((((........)))).(((((.......))))).....(((((.......))))))))))))....

The base pair distance between two secondary structures is given by the number of base pairs that have to be opened and closed to transform one structure into the other (as implemented in the RNAfold algorithm [18]). The Hamming distance between two secondary structures of the same length is given by the number of positions in which the two structures, aligned in standard bracket notation, differ.

Two relevant macroscopic quantities to characterize the state of the population are the average distance *d *to the target structure and the fraction *ρ *of structures in the population folding into the target structure. Due to the stochastic nature of evolution, both quantities fluctuate in time even after reaching an asymptotic regime. Therefore, within this regime, we perform averages over long time intervals (and different realizations, starting from distinct initial RNA populations). The obtained mean values are denoted by d¯
 MathType@MTEF@5@5@+=feaafiart1ev1aaatCvAUfKttLearuWrP9MDH5MBPbIqV92AaeXatLxBI9gBaebbnrfifHhDYfgasaacH8akY=wiFfYdH8Gipec8Eeeu0xXdbba9frFj0=OqFfea0dXdd9vqai=hGuQ8kuc9pgc9s8qqaq=dirpe0xb9q8qiLsFr0=vr0=vr0dc8meaabaqaciaacaGaaeqabaqabeGadaaakeaacuWGKbazgaqeaaaa@2E15@ and ρ¯
 MathType@MTEF@5@5@+=feaafiart1ev1aaatCvAUfKttLearuWrP9MDH5MBPbIqV92AaeXatLxBI9gBaebbnrfifHhDYfgasaacH8akY=wiFfYdH8Gipec8Eeeu0xXdbba9frFj0=OqFfea0dXdd9vqai=hGuQ8kuc9pgc9s8qqaq=dirpe0xb9q8qiLsFr0=vr0=vr0dc8meaabaqaciaacaGaaeqabaqabeGadaaakeaaiiGacuWFbpGCgaqeaaaa@2E8B@, respectively.

In order to quantify collective properties of the molecular ensemble, we calculate the *consensus sequence *of the population. The (virtual) consensus sequence is calculated by determining, for each position along the sequence, the most frequent type of nucleotide found within the population, i.e. "C", "A", "G" or "U". In real RNA molecular and viral quasispecies, the consensus sequence is obtained by means of population sequencing, and it does not necessarily correspond to any of the individual sequences present in the population.

It is straightforward to fold this sequence and obtain the *structure of the consensus sequence*, for which also its coincidence with the target structure can be determined. At each time step we count either one, corresponding to coincidence, or zero, otherwise. Averages over time (and realizations) of this binary variable yield ρ¯
 MathType@MTEF@5@5@+=feaafiart1ev1aaatCvAUfKttLearuWrP9MDH5MBPbIqV92AaeXatLxBI9gBaebbnrfifHhDYfgasaacH8akY=wiFfYdH8Gipec8Eeeu0xXdbba9frFj0=OqFfea0dXdd9vqai=hGuQ8kuc9pgc9s8qqaq=dirpe0xb9q8qiLsFr0=vr0=vr0dc8meaabaqaciaacaGaaeqabaqabeGadaaakeaaiiGacuWFbpGCgaqeaaaa@2E8B@_*C*_, which corresponds to the probability that, at a randomly chosen time step, the structure of the consensus sequence coincides with the target structure.

**Figure 1:**
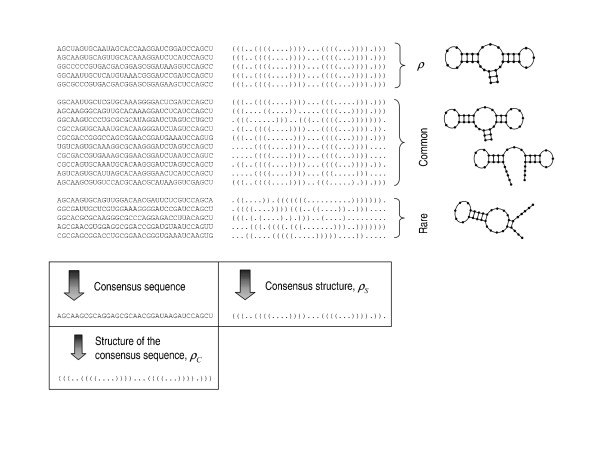
**Heterogeneity of sequences and structures, and quantities related**. Examples of RNA sequences (written in 5'-3' orientation) and their corresponding structures in bracket notation at the statistically stationary state of a numerical simulation with *N *= 602, *n *= 35, *μ *= 0.0325. The probability that a sequence folds into the target structure (a hammerhead structure, represented in the upper right corner) is ρ¯
 MathType@MTEF@5@5@+=feaafiart1ev1aaatCvAUfKttLearuWrP9MDH5MBPbIqV92AaeXatLxBI9gBaebbnrfifHhDYfgasaacH8akY=wiFfYdH8Gipec8Eeeu0xXdbba9frFj0=OqFfea0dXdd9vqai=hGuQ8kuc9pgc9s8qqaq=dirpe0xb9q8qiLsFr0=vr0=vr0dc8meaabaqaciaacaGaaeqabaqabeGadaaakeaaiiGacuWFbpGCgaqeaaaa@2E8B@ = 0.25. Other densities are ρ¯
 MathType@MTEF@5@5@+=feaafiart1ev1aaatCvAUfKttLearuWrP9MDH5MBPbIqV92AaeXatLxBI9gBaebbnrfifHhDYfgasaacH8akY=wiFfYdH8Gipec8Eeeu0xXdbba9frFj0=OqFfea0dXdd9vqai=hGuQ8kuc9pgc9s8qqaq=dirpe0xb9q8qiLsFr0=vr0=vr0dc8meaabaqaciaacaGaaeqabaqabeGadaaakeaaiiGacuWFbpGCgaqeaaaa@2E8B@_*C *_= 0.85, ρ¯
 MathType@MTEF@5@5@+=feaafiart1ev1aaatCvAUfKttLearuWrP9MDH5MBPbIqV92AaeXatLxBI9gBaebbnrfifHhDYfgasaacH8akY=wiFfYdH8Gipec8Eeeu0xXdbba9frFj0=OqFfea0dXdd9vqai=hGuQ8kuc9pgc9s8qqaq=dirpe0xb9q8qiLsFr0=vr0=vr0dc8meaabaqaciaacaGaaeqabaqabeGadaaakeaaiiGacuWFbpGCgaqeaaaa@2E8B@_*S *_= 0.99, and ρ¯
 MathType@MTEF@5@5@+=feaafiart1ev1aaatCvAUfKttLearuWrP9MDH5MBPbIqV92AaeXatLxBI9gBaebbnrfifHhDYfgasaacH8akY=wiFfYdH8Gipec8Eeeu0xXdbba9frFj0=OqFfea0dXdd9vqai=hGuQ8kuc9pgc9s8qqaq=dirpe0xb9q8qiLsFr0=vr0=vr0dc8meaabaqaciaacaGaaeqabaqabeGadaaakeaaiiGacuWFbpGCgaqeaaaa@2E8B@_*M *_= 0.01. At the time step chosen, the structure of the consensus sequence and the consensus structure differ in one position, and the latter does not correspond to any real secondary RNA structure (a very rare event in the asymptotic regime, but shown here for illustration). The Hamming distance between the sequences shown and the consensus sequence varies from 8 to 15, and does not show any significant difference whether the sequence folds into the target structure or whether the corresponding structure is rare (last group). Despite a well defined global state where the population, as a whole, clearly contains all the information on the target structure, both the density of sequences folding into it and especially the density of the most abundant sequence present low values.

In analogy to the consensus sequence, we further define a *consensus structure*. The (virtual) consensus structure is calculated by determining, for each position along the chain, the most frequent structural state found within the population, i.e. unpaired ".", paired upstream "(" or paired downstream ")". Due to this definition, the consensus structure again does not necessarily represent a valid secondary structure of an RNA molecule. Averages over time (and realizations) of the coincidence between the consensus structure and the target structure yield the probability ρ¯
 MathType@MTEF@5@5@+=feaafiart1ev1aaatCvAUfKttLearuWrP9MDH5MBPbIqV92AaeXatLxBI9gBaebbnrfifHhDYfgasaacH8akY=wiFfYdH8Gipec8Eeeu0xXdbba9frFj0=OqFfea0dXdd9vqai=hGuQ8kuc9pgc9s8qqaq=dirpe0xb9q8qiLsFr0=vr0=vr0dc8meaabaqaciaacaGaaeqabaqabeGadaaakeaaiiGacuWFbpGCgaqeaaaa@2E8B@_*S*_. Finally, it is also possible to identify the structure of the most abundant sequence in the population, to which a density ρ¯
 MathType@MTEF@5@5@+=feaafiart1ev1aaatCvAUfKttLearuWrP9MDH5MBPbIqV92AaeXatLxBI9gBaebbnrfifHhDYfgasaacH8akY=wiFfYdH8Gipec8Eeeu0xXdbba9frFj0=OqFfea0dXdd9vqai=hGuQ8kuc9pgc9s8qqaq=dirpe0xb9q8qiLsFr0=vr0=vr0dc8meaabaqaciaacaGaaeqabaqabeGadaaakeaaiiGacuWFbpGCgaqeaaaa@2E8B@_*M *_is assigned. Figure 1 depicts a sample of sequences and structures obtained from a real computational run of the model, and the different densities defined.

The size of the population ranges from *N *= 150 to *N *= 60000 molecules, though most results have been obtained for *N *= 602 (one zeptomol). Standard deviations have been computed for all averaged quantities, although they are explicitly shown in the figures only when of particular interest.

The quantities introduced so far correspond to observables of the population at the statistically stationary state. Starting from a random initial condition, there is a transient time required to attain asymptotic values of those quantities. In order to ensure that the transient behavior is not included in averaged quantities, we check for (a) the generation at which the first molecule folding correctly into the target structure appears, *g*_*A*_, and (b) the generation at which the target structure becomes fixed in the population, *g*_*F*_.

### Numerical results

#### Statistically stationary state

After the initial transient time has elapsed, the density *ρ *only fluctuates in time around a constant value with a strength that decreases as the inverse of the square root of the population size *N*. Once this asymptotic regime is reached, averages over time and realizations are performed to yield the mean value ρ¯
 MathType@MTEF@5@5@+=feaafiart1ev1aaatCvAUfKttLearuWrP9MDH5MBPbIqV92AaeXatLxBI9gBaebbnrfifHhDYfgasaacH8akY=wiFfYdH8Gipec8Eeeu0xXdbba9frFj0=OqFfea0dXdd9vqai=hGuQ8kuc9pgc9s8qqaq=dirpe0xb9q8qiLsFr0=vr0=vr0dc8meaabaqaciaacaGaaeqabaqabeGadaaakeaaiiGacuWFbpGCgaqeaaaa@2E8B@ corresponding to a given mutation rate *μ*.

**Figure 2:**
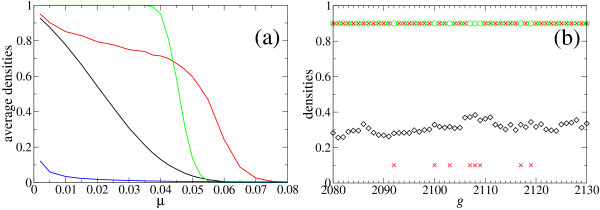
**Average values of population densities as function of *μ *and typical dynamical behavior**. (a) Values of the average density ρ¯
 MathType@MTEF@5@5@+=feaafiart1ev1aaatCvAUfKttLearuWrP9MDH5MBPbIqV92AaeXatLxBI9gBaebbnrfifHhDYfgasaacH8akY=wiFfYdH8Gipec8Eeeu0xXdbba9frFj0=OqFfea0dXdd9vqai=hGuQ8kuc9pgc9s8qqaq=dirpe0xb9q8qiLsFr0=vr0=vr0dc8meaabaqaciaacaGaaeqabaqabeGadaaakeaaiiGacuWFbpGCgaqeaaaa@2E8B@ (black curve) of sequences folding into the target structure (hairpin), of the probability ρ¯
 MathType@MTEF@5@5@+=feaafiart1ev1aaatCvAUfKttLearuWrP9MDH5MBPbIqV92AaeXatLxBI9gBaebbnrfifHhDYfgasaacH8akY=wiFfYdH8Gipec8Eeeu0xXdbba9frFj0=OqFfea0dXdd9vqai=hGuQ8kuc9pgc9s8qqaq=dirpe0xb9q8qiLsFr0=vr0=vr0dc8meaabaqaciaacaGaaeqabaqabeGadaaakeaaiiGacuWFbpGCgaqeaaaa@2E8B@_*C *_(red line) that the consensus sequence folds into the target structure, of the probability ρ¯
 MathType@MTEF@5@5@+=feaafiart1ev1aaatCvAUfKttLearuWrP9MDH5MBPbIqV92AaeXatLxBI9gBaebbnrfifHhDYfgasaacH8akY=wiFfYdH8Gipec8Eeeu0xXdbba9frFj0=OqFfea0dXdd9vqai=hGuQ8kuc9pgc9s8qqaq=dirpe0xb9q8qiLsFr0=vr0=vr0dc8meaabaqaciaacaGaaeqabaqabeGadaaakeaaiiGacuWFbpGCgaqeaaaa@2E8B@_*S *_(green line) that the consensus structure coincides with the target structure, and of the fraction ρ¯
 MathType@MTEF@5@5@+=feaafiart1ev1aaatCvAUfKttLearuWrP9MDH5MBPbIqV92AaeXatLxBI9gBaebbnrfifHhDYfgasaacH8akY=wiFfYdH8Gipec8Eeeu0xXdbba9frFj0=OqFfea0dXdd9vqai=hGuQ8kuc9pgc9s8qqaq=dirpe0xb9q8qiLsFr0=vr0=vr0dc8meaabaqaciaacaGaaeqabaqabeGadaaakeaaiiGacuWFbpGCgaqeaaaa@2E8B@_*M *_(blue line) of the most abundant sequence in the population. Population size is *N *= 602; averages over 4000 generations and 25 realizations have been performed. (b) Temporal dynamics (interval of 50 generations starting at generation 2080 after initialization of a single run) of *ρ *(black diamonds), *ρ*_*C *_(red crosses), and *ρ*_*S *_(green circles) for *μ *= 0.030. Average values are ρ¯
 MathType@MTEF@5@5@+=feaafiart1ev1aaatCvAUfKttLearuWrP9MDH5MBPbIqV92AaeXatLxBI9gBaebbnrfifHhDYfgasaacH8akY=wiFfYdH8Gipec8Eeeu0xXdbba9frFj0=OqFfea0dXdd9vqai=hGuQ8kuc9pgc9s8qqaq=dirpe0xb9q8qiLsFr0=vr0=vr0dc8meaabaqaciaacaGaaeqabaqabeGadaaakeaaiiGacuWFbpGCgaqeaaaa@2E8B@ = 0.323, ρ¯
 MathType@MTEF@5@5@+=feaafiart1ev1aaatCvAUfKttLearuWrP9MDH5MBPbIqV92AaeXatLxBI9gBaebbnrfifHhDYfgasaacH8akY=wiFfYdH8Gipec8Eeeu0xXdbba9frFj0=OqFfea0dXdd9vqai=hGuQ8kuc9pgc9s8qqaq=dirpe0xb9q8qiLsFr0=vr0=vr0dc8meaabaqaciaacaGaaeqabaqabeGadaaakeaaiiGacuWFbpGCgaqeaaaa@2E8B@_*C *_= 0.749, ρ¯
 MathType@MTEF@5@5@+=feaafiart1ev1aaatCvAUfKttLearuWrP9MDH5MBPbIqV92AaeXatLxBI9gBaebbnrfifHhDYfgasaacH8akY=wiFfYdH8Gipec8Eeeu0xXdbba9frFj0=OqFfea0dXdd9vqai=hGuQ8kuc9pgc9s8qqaq=dirpe0xb9q8qiLsFr0=vr0=vr0dc8meaabaqaciaacaGaaeqabaqabeGadaaakeaaiiGacuWFbpGCgaqeaaaa@2E8B@_*S *_= 1.0. For better visualization of the values of *ρ*_*C *_and *ρ*_*S*_, the values corresponding to 1 are drawn on the line 0.9 and the values corresponding to 0 are drawn on the line 0.1.

Same averages are performed over the other densities defined. Figure 2(a) shows average values ρ¯
 MathType@MTEF@5@5@+=feaafiart1ev1aaatCvAUfKttLearuWrP9MDH5MBPbIqV92AaeXatLxBI9gBaebbnrfifHhDYfgasaacH8akY=wiFfYdH8Gipec8Eeeu0xXdbba9frFj0=OqFfea0dXdd9vqai=hGuQ8kuc9pgc9s8qqaq=dirpe0xb9q8qiLsFr0=vr0=vr0dc8meaabaqaciaacaGaaeqabaqabeGadaaakeaaiiGacuWFbpGCgaqeaaaa@2E8B@, ρ¯
 MathType@MTEF@5@5@+=feaafiart1ev1aaatCvAUfKttLearuWrP9MDH5MBPbIqV92AaeXatLxBI9gBaebbnrfifHhDYfgasaacH8akY=wiFfYdH8Gipec8Eeeu0xXdbba9frFj0=OqFfea0dXdd9vqai=hGuQ8kuc9pgc9s8qqaq=dirpe0xb9q8qiLsFr0=vr0=vr0dc8meaabaqaciaacaGaaeqabaqabeGadaaakeaaiiGacuWFbpGCgaqeaaaa@2E8B@_*S*_, ρ¯
 MathType@MTEF@5@5@+=feaafiart1ev1aaatCvAUfKttLearuWrP9MDH5MBPbIqV92AaeXatLxBI9gBaebbnrfifHhDYfgasaacH8akY=wiFfYdH8Gipec8Eeeu0xXdbba9frFj0=OqFfea0dXdd9vqai=hGuQ8kuc9pgc9s8qqaq=dirpe0xb9q8qiLsFr0=vr0=vr0dc8meaabaqaciaacaGaaeqabaqabeGadaaakeaaiiGacuWFbpGCgaqeaaaa@2E8B@_*C*_, and ρ¯
 MathType@MTEF@5@5@+=feaafiart1ev1aaatCvAUfKttLearuWrP9MDH5MBPbIqV92AaeXatLxBI9gBaebbnrfifHhDYfgasaacH8akY=wiFfYdH8Gipec8Eeeu0xXdbba9frFj0=OqFfea0dXdd9vqai=hGuQ8kuc9pgc9s8qqaq=dirpe0xb9q8qiLsFr0=vr0=vr0dc8meaabaqaciaacaGaaeqabaqabeGadaaakeaaiiGacuWFbpGCgaqeaaaa@2E8B@_*M *_as a function of *μ*. Since, for most cases, the transient is not longer than a few hundred generations, the first 2000 generations are skipped and the average is performed over 4000 generations and 25 realizations.

For values of the mutation rate below the phenotypic error threshold, the density ρ¯
 MathType@MTEF@5@5@+=feaafiart1ev1aaatCvAUfKttLearuWrP9MDH5MBPbIqV92AaeXatLxBI9gBaebbnrfifHhDYfgasaacH8akY=wiFfYdH8Gipec8Eeeu0xXdbba9frFj0=OqFfea0dXdd9vqai=hGuQ8kuc9pgc9s8qqaq=dirpe0xb9q8qiLsFr0=vr0=vr0dc8meaabaqaciaacaGaaeqabaqabeGadaaakeaaiiGacuWFbpGCgaqeaaaa@2E8B@ always yields finite values. The asymptotic value of the phenotypic error threshold is only obtained in the limit of population size *N *→ ∞. At finite population size, the error threshold is shifted towards lower replication error rates [19]. In this work, by phenotypic error threshold we mean the value of *μ *at which ρ¯
 MathType@MTEF@5@5@+=feaafiart1ev1aaatCvAUfKttLearuWrP9MDH5MBPbIqV92AaeXatLxBI9gBaebbnrfifHhDYfgasaacH8akY=wiFfYdH8Gipec8Eeeu0xXdbba9frFj0=OqFfea0dXdd9vqai=hGuQ8kuc9pgc9s8qqaq=dirpe0xb9q8qiLsFr0=vr0=vr0dc8meaabaqaciaacaGaaeqabaqabeGadaaakeaaiiGacuWFbpGCgaqeaaaa@2E8B@ ≃ 0. A precise estimation of the error threshold is not necessary for the qualitative effects here described. As *μ *increases, ρ¯
 MathType@MTEF@5@5@+=feaafiart1ev1aaatCvAUfKttLearuWrP9MDH5MBPbIqV92AaeXatLxBI9gBaebbnrfifHhDYfgasaacH8akY=wiFfYdH8Gipec8Eeeu0xXdbba9frFj0=OqFfea0dXdd9vqai=hGuQ8kuc9pgc9s8qqaq=dirpe0xb9q8qiLsFr0=vr0=vr0dc8meaabaqaciaacaGaaeqabaqabeGadaaakeaaiiGacuWFbpGCgaqeaaaa@2E8B@ decreases monotonously. If the mutation rate is too large, the phenotypic error threshold *μ*_*c *_is crossed and the population cannot evolve successfully towards the target structure. Above *μ*_*c *_the target structure is only present due to stochastic effects, and ρ¯
 MathType@MTEF@5@5@+=feaafiart1ev1aaatCvAUfKttLearuWrP9MDH5MBPbIqV92AaeXatLxBI9gBaebbnrfifHhDYfgasaacH8akY=wiFfYdH8Gipec8Eeeu0xXdbba9frFj0=OqFfea0dXdd9vqai=hGuQ8kuc9pgc9s8qqaq=dirpe0xb9q8qiLsFr0=vr0=vr0dc8meaabaqaciaacaGaaeqabaqabeGadaaakeaaiiGacuWFbpGCgaqeaaaa@2E8B@ ≪ 1 (for an estimation of a lower bound to the probability that the target structure appears in a population of random sequences, see [20]). However, ρ¯
 MathType@MTEF@5@5@+=feaafiart1ev1aaatCvAUfKttLearuWrP9MDH5MBPbIqV92AaeXatLxBI9gBaebbnrfifHhDYfgasaacH8akY=wiFfYdH8Gipec8Eeeu0xXdbba9frFj0=OqFfea0dXdd9vqai=hGuQ8kuc9pgc9s8qqaq=dirpe0xb9q8qiLsFr0=vr0=vr0dc8meaabaqaciaacaGaaeqabaqabeGadaaakeaaiiGacuWFbpGCgaqeaaaa@2E8B@ vanishes strictly at (and hence above) the error threshold, i.e. lim⁡μ→μc ρ¯→0
 MathType@MTEF@5@5@+=feaafiart1ev1aaatCvAUfKttLearuWrP9MDH5MBPbIqV92AaeXatLxBI9gBaebbnrfifHhDYfgasaacH8akY=wiFfYdH8Gipec8Eeeu0xXdbba9frFj0=OqFfea0dXdd9vqai=hGuQ8kuc9pgc9s8qqaq=dirpe0xb9q8qiLsFr0=vr0=vr0dc8meaabaqaciaacaGaaeqabaqabeGadaaakeaacyGGSbaBcqGGPbqAcqGGTbqBdaWgaaWcbaacciGae8hVd0MaeyOKH4Qae8hVd02aaSbaaWqaaiabdogaJbqabaaaleqaaOGaf8xWdiNbaebacqGHsgIRcqaIWaamaaa@3C90@, only in the limit *n *→ ∞, *N *→ ∞. While ρ¯
 MathType@MTEF@5@5@+=feaafiart1ev1aaatCvAUfKttLearuWrP9MDH5MBPbIqV92AaeXatLxBI9gBaebbnrfifHhDYfgasaacH8akY=wiFfYdH8Gipec8Eeeu0xXdbba9frFj0=OqFfea0dXdd9vqai=hGuQ8kuc9pgc9s8qqaq=dirpe0xb9q8qiLsFr0=vr0=vr0dc8meaabaqaciaacaGaaeqabaqabeGadaaakeaaiiGacuWFbpGCgaqeaaaa@2E8B@ decreases smoothly over a large range of *μ*, the situation is different for the density of the consensus structure ρ¯
 MathType@MTEF@5@5@+=feaafiart1ev1aaatCvAUfKttLearuWrP9MDH5MBPbIqV92AaeXatLxBI9gBaebbnrfifHhDYfgasaacH8akY=wiFfYdH8Gipec8Eeeu0xXdbba9frFj0=OqFfea0dXdd9vqai=hGuQ8kuc9pgc9s8qqaq=dirpe0xb9q8qiLsFr0=vr0=vr0dc8meaabaqaciaacaGaaeqabaqabeGadaaakeaaiiGacuWFbpGCgaqeaaaa@2E8B@_*S*_, which is essentially one below *μ*_*c *_and jumps to zero above the threshold value. The apparently smooth transition resulting from the numerical simulations is a finite size effect. Actually, we expect that the transition experienced by ρ¯
 MathType@MTEF@5@5@+=feaafiart1ev1aaatCvAUfKttLearuWrP9MDH5MBPbIqV92AaeXatLxBI9gBaebbnrfifHhDYfgasaacH8akY=wiFfYdH8Gipec8Eeeu0xXdbba9frFj0=OqFfea0dXdd9vqai=hGuQ8kuc9pgc9s8qqaq=dirpe0xb9q8qiLsFr0=vr0=vr0dc8meaabaqaciaacaGaaeqabaqabeGadaaakeaaiiGacuWFbpGCgaqeaaaa@2E8B@_*S *_is discontinuous and analogous to the disintegration of information described for quasispecies at the genotypic error threshold [21,22]. It is interesting that, close to the threshold, information on the phenotype is a collective property: it is clearly recovered when averages over the population are performed, but phenotype is delocalized, in the sense that only a few sequences truly fold into the correct target structure.

The density of the structure of the consensus sequence ρ¯
 MathType@MTEF@5@5@+=feaafiart1ev1aaatCvAUfKttLearuWrP9MDH5MBPbIqV92AaeXatLxBI9gBaebbnrfifHhDYfgasaacH8akY=wiFfYdH8Gipec8Eeeu0xXdbba9frFj0=OqFfea0dXdd9vqai=hGuQ8kuc9pgc9s8qqaq=dirpe0xb9q8qiLsFr0=vr0=vr0dc8meaabaqaciaacaGaaeqabaqabeGadaaakeaaiiGacuWFbpGCgaqeaaaa@2E8B@_*C *_behaves differently from ρ¯
 MathType@MTEF@5@5@+=feaafiart1ev1aaatCvAUfKttLearuWrP9MDH5MBPbIqV92AaeXatLxBI9gBaebbnrfifHhDYfgasaacH8akY=wiFfYdH8Gipec8Eeeu0xXdbba9frFj0=OqFfea0dXdd9vqai=hGuQ8kuc9pgc9s8qqaq=dirpe0xb9q8qiLsFr0=vr0=vr0dc8meaabaqaciaacaGaaeqabaqabeGadaaakeaaiiGacuWFbpGCgaqeaaaa@2E8B@ and ρ¯
 MathType@MTEF@5@5@+=feaafiart1ev1aaatCvAUfKttLearuWrP9MDH5MBPbIqV92AaeXatLxBI9gBaebbnrfifHhDYfgasaacH8akY=wiFfYdH8Gipec8Eeeu0xXdbba9frFj0=OqFfea0dXdd9vqai=hGuQ8kuc9pgc9s8qqaq=dirpe0xb9q8qiLsFr0=vr0=vr0dc8meaabaqaciaacaGaaeqabaqabeGadaaakeaaiiGacuWFbpGCgaqeaaaa@2E8B@_*S*_. Although it decreases smoothly as *μ *increases, it keeps relatively high values even for *μ *> *μ*_*c*_.

The frequency ρ¯
 MathType@MTEF@5@5@+=feaafiart1ev1aaatCvAUfKttLearuWrP9MDH5MBPbIqV92AaeXatLxBI9gBaebbnrfifHhDYfgasaacH8akY=wiFfYdH8Gipec8Eeeu0xXdbba9frFj0=OqFfea0dXdd9vqai=hGuQ8kuc9pgc9s8qqaq=dirpe0xb9q8qiLsFr0=vr0=vr0dc8meaabaqaciaacaGaaeqabaqabeGadaaakeaaiiGacuWFbpGCgaqeaaaa@2E8B@_*M *_of the most abundant sequence displays relatively low values for all mutation rates, except in the limit *μ *→ 0 where the population becomes homogeneous. In the present case, and since mutation and selection operate on different levels, it is not surprising to find that many different sequences fold into the same structure. This multiplicity is a consequence of the degeneration of the sequence-structure map. Numerical results not displayed here show that ρ¯
 MathType@MTEF@5@5@+=feaafiart1ev1aaatCvAUfKttLearuWrP9MDH5MBPbIqV92AaeXatLxBI9gBaebbnrfifHhDYfgasaacH8akY=wiFfYdH8Gipec8Eeeu0xXdbba9frFj0=OqFfea0dXdd9vqai=hGuQ8kuc9pgc9s8qqaq=dirpe0xb9q8qiLsFr0=vr0=vr0dc8meaabaqaciaacaGaaeqabaqabeGadaaakeaaiiGacuWFbpGCgaqeaaaa@2E8B@_*M *_decreases as the population size *N *increases, since a larger population is able to explore a proportionally larger fraction of the sequence space. A conclusion of this observation is that, for most *μ *values, the most abundant sequence does not give any relevant information on the structure and composition of the population. Therefore, from a functional point of view, we cannot think of quasispecies as ensembles organized around a typical sequence. The consensus sequence can be better described as a center of gravity of the population [22].

Figure 2(b) displays a short time interval illustrating the typical behavior of the three densities, *ρ*, *ρ*_*S*_, and *ρ*_*C *_in the asymptotic regime for the particular case *μ *= 0.03. The values of *ρ *fluctuate slightly around its average value ρ¯
 MathType@MTEF@5@5@+=feaafiart1ev1aaatCvAUfKttLearuWrP9MDH5MBPbIqV92AaeXatLxBI9gBaebbnrfifHhDYfgasaacH8akY=wiFfYdH8Gipec8Eeeu0xXdbba9frFj0=OqFfea0dXdd9vqai=hGuQ8kuc9pgc9s8qqaq=dirpe0xb9q8qiLsFr0=vr0=vr0dc8meaabaqaciaacaGaaeqabaqabeGadaaakeaaiiGacuWFbpGCgaqeaaaa@2E8B@ = 0.323. Nevertheless, although only a relatively small fraction of sequences fold into the target structure, the consensus structure matches practically always the target structure: ρ¯
 MathType@MTEF@5@5@+=feaafiart1ev1aaatCvAUfKttLearuWrP9MDH5MBPbIqV92AaeXatLxBI9gBaebbnrfifHhDYfgasaacH8akY=wiFfYdH8Gipec8Eeeu0xXdbba9frFj0=OqFfea0dXdd9vqai=hGuQ8kuc9pgc9s8qqaq=dirpe0xb9q8qiLsFr0=vr0=vr0dc8meaabaqaciaacaGaaeqabaqabeGadaaakeaaiiGacuWFbpGCgaqeaaaa@2E8B@_*S *_= 1.0. The structure of the consensus sequence is equal to the target structure in most generations, but not in all of them, in agreement with its average value ρ¯
 MathType@MTEF@5@5@+=feaafiart1ev1aaatCvAUfKttLearuWrP9MDH5MBPbIqV92AaeXatLxBI9gBaebbnrfifHhDYfgasaacH8akY=wiFfYdH8Gipec8Eeeu0xXdbba9frFj0=OqFfea0dXdd9vqai=hGuQ8kuc9pgc9s8qqaq=dirpe0xb9q8qiLsFr0=vr0=vr0dc8meaabaqaciaacaGaaeqabaqabeGadaaakeaaiiGacuWFbpGCgaqeaaaa@2E8B@_*C *_= 0.749. The coincidences between the structure of the consensus sequence or the consensus structure with the target structure is intermittent in time due to fluctuations in the composition of the ensemble of sequences. Examination of long time series show that if at a given generation only relatively few sequences fold into the target structure, at the same time the probability increases that the structure of the consensus sequence does not coincide with the target structure.

**Figure 3:**
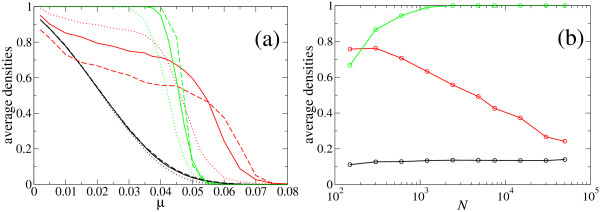
**Densities as a function of the mutation rate *μ *and the population size *N***. (a) Curves for ρ¯
 MathType@MTEF@5@5@+=feaafiart1ev1aaatCvAUfKttLearuWrP9MDH5MBPbIqV92AaeXatLxBI9gBaebbnrfifHhDYfgasaacH8akY=wiFfYdH8Gipec8Eeeu0xXdbba9frFj0=OqFfea0dXdd9vqai=hGuQ8kuc9pgc9s8qqaq=dirpe0xb9q8qiLsFr0=vr0=vr0dc8meaabaqaciaacaGaaeqabaqabeGadaaakeaaiiGacuWFbpGCgaqeaaaa@2E8B@ (black lines), ρ¯
 MathType@MTEF@5@5@+=feaafiart1ev1aaatCvAUfKttLearuWrP9MDH5MBPbIqV92AaeXatLxBI9gBaebbnrfifHhDYfgasaacH8akY=wiFfYdH8Gipec8Eeeu0xXdbba9frFj0=OqFfea0dXdd9vqai=hGuQ8kuc9pgc9s8qqaq=dirpe0xb9q8qiLsFr0=vr0=vr0dc8meaabaqaciaacaGaaeqabaqabeGadaaakeaaiiGacuWFbpGCgaqeaaaa@2E8B@_*C *_(red lines), and ρ¯
 MathType@MTEF@5@5@+=feaafiart1ev1aaatCvAUfKttLearuWrP9MDH5MBPbIqV92AaeXatLxBI9gBaebbnrfifHhDYfgasaacH8akY=wiFfYdH8Gipec8Eeeu0xXdbba9frFj0=OqFfea0dXdd9vqai=hGuQ8kuc9pgc9s8qqaq=dirpe0xb9q8qiLsFr0=vr0=vr0dc8meaabaqaciaacaGaaeqabaqabeGadaaakeaaiiGacuWFbpGCgaqeaaaa@2E8B@_*S *_(green lines) as a function of the mutation rate for different values of the population size *N*. Dotted lines denote *N *= 150, solid lines *N *= 602, and dashed lines *N *= 2408. The density of correctly folded sequences ρ¯
 MathType@MTEF@5@5@+=feaafiart1ev1aaatCvAUfKttLearuWrP9MDH5MBPbIqV92AaeXatLxBI9gBaebbnrfifHhDYfgasaacH8akY=wiFfYdH8Gipec8Eeeu0xXdbba9frFj0=OqFfea0dXdd9vqai=hGuQ8kuc9pgc9s8qqaq=dirpe0xb9q8qiLsFr0=vr0=vr0dc8meaabaqaciaacaGaaeqabaqabeGadaaakeaaiiGacuWFbpGCgaqeaaaa@2E8B@ and the probability ρ¯
 MathType@MTEF@5@5@+=feaafiart1ev1aaatCvAUfKttLearuWrP9MDH5MBPbIqV92AaeXatLxBI9gBaebbnrfifHhDYfgasaacH8akY=wiFfYdH8Gipec8Eeeu0xXdbba9frFj0=OqFfea0dXdd9vqai=hGuQ8kuc9pgc9s8qqaq=dirpe0xb9q8qiLsFr0=vr0=vr0dc8meaabaqaciaacaGaaeqabaqabeGadaaakeaaiiGacuWFbpGCgaqeaaaa@2E8B@_*S *_that the consensus structure coincides with the target structure converge to limit curves as *N *increases. The probability ρ¯
 MathType@MTEF@5@5@+=feaafiart1ev1aaatCvAUfKttLearuWrP9MDH5MBPbIqV92AaeXatLxBI9gBaebbnrfifHhDYfgasaacH8akY=wiFfYdH8Gipec8Eeeu0xXdbba9frFj0=OqFfea0dXdd9vqai=hGuQ8kuc9pgc9s8qqaq=dirpe0xb9q8qiLsFr0=vr0=vr0dc8meaabaqaciaacaGaaeqabaqabeGadaaakeaaiiGacuWFbpGCgaqeaaaa@2E8B@_*C *_depends strongly on *N*. (b) Dependence of ρ¯
 MathType@MTEF@5@5@+=feaafiart1ev1aaatCvAUfKttLearuWrP9MDH5MBPbIqV92AaeXatLxBI9gBaebbnrfifHhDYfgasaacH8akY=wiFfYdH8Gipec8Eeeu0xXdbba9frFj0=OqFfea0dXdd9vqai=hGuQ8kuc9pgc9s8qqaq=dirpe0xb9q8qiLsFr0=vr0=vr0dc8meaabaqaciaacaGaaeqabaqabeGadaaakeaaiiGacuWFbpGCgaqeaaaa@2E8B@, ρ¯
 MathType@MTEF@5@5@+=feaafiart1ev1aaatCvAUfKttLearuWrP9MDH5MBPbIqV92AaeXatLxBI9gBaebbnrfifHhDYfgasaacH8akY=wiFfYdH8Gipec8Eeeu0xXdbba9frFj0=OqFfea0dXdd9vqai=hGuQ8kuc9pgc9s8qqaq=dirpe0xb9q8qiLsFr0=vr0=vr0dc8meaabaqaciaacaGaaeqabaqabeGadaaakeaaiiGacuWFbpGCgaqeaaaa@2E8B@_*C*_, and ρ¯
 MathType@MTEF@5@5@+=feaafiart1ev1aaatCvAUfKttLearuWrP9MDH5MBPbIqV92AaeXatLxBI9gBaebbnrfifHhDYfgasaacH8akY=wiFfYdH8Gipec8Eeeu0xXdbba9frFj0=OqFfea0dXdd9vqai=hGuQ8kuc9pgc9s8qqaq=dirpe0xb9q8qiLsFr0=vr0=vr0dc8meaabaqaciaacaGaaeqabaqabeGadaaakeaaiiGacuWFbpGCgaqeaaaa@2E8B@_*S *_with *N*, quantitatively illustrating the behaviors described in (a), for the specific mutation rate *μ *= 0.04. Averages have been performed over 4000 generations and 25 realizations.

Average curves showing the dependence of the densities on *N *are represented in Figure 3. In Fig. 3(a) we display ρ¯
 MathType@MTEF@5@5@+=feaafiart1ev1aaatCvAUfKttLearuWrP9MDH5MBPbIqV92AaeXatLxBI9gBaebbnrfifHhDYfgasaacH8akY=wiFfYdH8Gipec8Eeeu0xXdbba9frFj0=OqFfea0dXdd9vqai=hGuQ8kuc9pgc9s8qqaq=dirpe0xb9q8qiLsFr0=vr0=vr0dc8meaabaqaciaacaGaaeqabaqabeGadaaakeaaiiGacuWFbpGCgaqeaaaa@2E8B@, ρ¯
 MathType@MTEF@5@5@+=feaafiart1ev1aaatCvAUfKttLearuWrP9MDH5MBPbIqV92AaeXatLxBI9gBaebbnrfifHhDYfgasaacH8akY=wiFfYdH8Gipec8Eeeu0xXdbba9frFj0=OqFfea0dXdd9vqai=hGuQ8kuc9pgc9s8qqaq=dirpe0xb9q8qiLsFr0=vr0=vr0dc8meaabaqaciaacaGaaeqabaqabeGadaaakeaaiiGacuWFbpGCgaqeaaaa@2E8B@_*S*_, and ρ¯
 MathType@MTEF@5@5@+=feaafiart1ev1aaatCvAUfKttLearuWrP9MDH5MBPbIqV92AaeXatLxBI9gBaebbnrfifHhDYfgasaacH8akY=wiFfYdH8Gipec8Eeeu0xXdbba9frFj0=OqFfea0dXdd9vqai=hGuQ8kuc9pgc9s8qqaq=dirpe0xb9q8qiLsFr0=vr0=vr0dc8meaabaqaciaacaGaaeqabaqabeGadaaakeaaiiGacuWFbpGCgaqeaaaa@2E8B@_*C *_as a function of *μ *for three different values of *N*. We observe the weak dependence of ρ¯
 MathType@MTEF@5@5@+=feaafiart1ev1aaatCvAUfKttLearuWrP9MDH5MBPbIqV92AaeXatLxBI9gBaebbnrfifHhDYfgasaacH8akY=wiFfYdH8Gipec8Eeeu0xXdbba9frFj0=OqFfea0dXdd9vqai=hGuQ8kuc9pgc9s8qqaq=dirpe0xb9q8qiLsFr0=vr0=vr0dc8meaabaqaciaacaGaaeqabaqabeGadaaakeaaiiGacuWFbpGCgaqeaaaa@2E8B@ on the population size and a fast convergence to its asymptotic value, the convergence of ρ¯
 MathType@MTEF@5@5@+=feaafiart1ev1aaatCvAUfKttLearuWrP9MDH5MBPbIqV92AaeXatLxBI9gBaebbnrfifHhDYfgasaacH8akY=wiFfYdH8Gipec8Eeeu0xXdbba9frFj0=OqFfea0dXdd9vqai=hGuQ8kuc9pgc9s8qqaq=dirpe0xb9q8qiLsFr0=vr0=vr0dc8meaabaqaciaacaGaaeqabaqabeGadaaakeaaiiGacuWFbpGCgaqeaaaa@2E8B@_*S *_to a step function at *μ *= *μ*_*c*_, and the large variation of ρ¯
 MathType@MTEF@5@5@+=feaafiart1ev1aaatCvAUfKttLearuWrP9MDH5MBPbIqV92AaeXatLxBI9gBaebbnrfifHhDYfgasaacH8akY=wiFfYdH8Gipec8Eeeu0xXdbba9frFj0=OqFfea0dXdd9vqai=hGuQ8kuc9pgc9s8qqaq=dirpe0xb9q8qiLsFr0=vr0=vr0dc8meaabaqaciaacaGaaeqabaqabeGadaaakeaaiiGacuWFbpGCgaqeaaaa@2E8B@_*C *_as *N *changes. The strong dependence of ρ¯
 MathType@MTEF@5@5@+=feaafiart1ev1aaatCvAUfKttLearuWrP9MDH5MBPbIqV92AaeXatLxBI9gBaebbnrfifHhDYfgasaacH8akY=wiFfYdH8Gipec8Eeeu0xXdbba9frFj0=OqFfea0dXdd9vqai=hGuQ8kuc9pgc9s8qqaq=dirpe0xb9q8qiLsFr0=vr0=vr0dc8meaabaqaciaacaGaaeqabaqabeGadaaakeaaiiGacuWFbpGCgaqeaaaa@2E8B@_*C *_on *N *limits the practical use of ρ¯
 MathType@MTEF@5@5@+=feaafiart1ev1aaatCvAUfKttLearuWrP9MDH5MBPbIqV92AaeXatLxBI9gBaebbnrfifHhDYfgasaacH8akY=wiFfYdH8Gipec8Eeeu0xXdbba9frFj0=OqFfea0dXdd9vqai=hGuQ8kuc9pgc9s8qqaq=dirpe0xb9q8qiLsFr0=vr0=vr0dc8meaabaqaciaacaGaaeqabaqabeGadaaakeaaiiGacuWFbpGCgaqeaaaa@2E8B@_*C *_as a quantity to describe the evolutionary state of the population. The trend of each quantity is illustrated in Fig. 3(b) for a particular value of the mutation rate.

**Figure 4:**
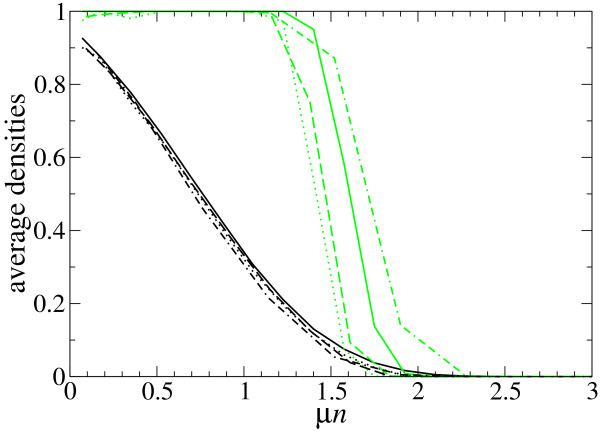
**Relevant densities for different target structures**. Dependence of ρ¯
 MathType@MTEF@5@5@+=feaafiart1ev1aaatCvAUfKttLearuWrP9MDH5MBPbIqV92AaeXatLxBI9gBaebbnrfifHhDYfgasaacH8akY=wiFfYdH8Gipec8Eeeu0xXdbba9frFj0=OqFfea0dXdd9vqai=hGuQ8kuc9pgc9s8qqaq=dirpe0xb9q8qiLsFr0=vr0=vr0dc8meaabaqaciaacaGaaeqabaqabeGadaaakeaaiiGacuWFbpGCgaqeaaaa@2E8B@ (black lines) and ρ¯
 MathType@MTEF@5@5@+=feaafiart1ev1aaatCvAUfKttLearuWrP9MDH5MBPbIqV92AaeXatLxBI9gBaebbnrfifHhDYfgasaacH8akY=wiFfYdH8Gipec8Eeeu0xXdbba9frFj0=OqFfea0dXdd9vqai=hGuQ8kuc9pgc9s8qqaq=dirpe0xb9q8qiLsFr0=vr0=vr0dc8meaabaqaciaacaGaaeqabaqabeGadaaakeaaiiGacuWFbpGCgaqeaaaa@2E8B@_*S *_(green lines) on the mutation rate *μ *and the length *n *of the target structure. The curves correspond to the hairpin structure (solid line), hammerhead structure (dotted line), 3-stem-loop structure (dashed line), model tRNA structure (dotted-dashed line), whose structures are specified in Table 1. The curves for ρ¯
 MathType@MTEF@5@5@+=feaafiart1ev1aaatCvAUfKttLearuWrP9MDH5MBPbIqV92AaeXatLxBI9gBaebbnrfifHhDYfgasaacH8akY=wiFfYdH8Gipec8Eeeu0xXdbba9frFj0=OqFfea0dXdd9vqai=hGuQ8kuc9pgc9s8qqaq=dirpe0xb9q8qiLsFr0=vr0=vr0dc8meaabaqaciaacaGaaeqabaqabeGadaaakeaaiiGacuWFbpGCgaqeaaaa@2E8B@ almost coincide when the dependent variable is scaled as *μn*. Population size is *N *= 602; averages over 4000 generations and 25 realizations have been performed.

Our study of the dependence of the densities in the asymptoticy state with the model parameters is completed by analyzing the role played by different target secondary structures. This involves not only the dependence on the sequence length *n*, but also on the particular shape used as target. To this end, we use two populations evolving towards a hairpin-like structure and towards a hammerhead structure, both of length *n *= 35, as well as two populations evolving towards a 3-stem loop (*n *= 46) and a model tRNA (*n *= 76), respectively (see Table 1). The different densities computed can be compared if the product *μn *is used as independent variable, instead of the bare mutation rate (Figure 4). In fact, we observe that the different curves for ρ¯
 MathType@MTEF@5@5@+=feaafiart1ev1aaatCvAUfKttLearuWrP9MDH5MBPbIqV92AaeXatLxBI9gBaebbnrfifHhDYfgasaacH8akY=wiFfYdH8Gipec8Eeeu0xXdbba9frFj0=OqFfea0dXdd9vqai=hGuQ8kuc9pgc9s8qqaq=dirpe0xb9q8qiLsFr0=vr0=vr0dc8meaabaqaciaacaGaaeqabaqabeGadaaakeaaiiGacuWFbpGCgaqeaaaa@2E8B@ almost coincide, though differences are genuine and not due to statistical errors.

Further, the four curves for ρ¯
 MathType@MTEF@5@5@+=feaafiart1ev1aaatCvAUfKttLearuWrP9MDH5MBPbIqV92AaeXatLxBI9gBaebbnrfifHhDYfgasaacH8akY=wiFfYdH8Gipec8Eeeu0xXdbba9frFj0=OqFfea0dXdd9vqai=hGuQ8kuc9pgc9s8qqaq=dirpe0xb9q8qiLsFr0=vr0=vr0dc8meaabaqaciaacaGaaeqabaqabeGadaaakeaaiiGacuWFbpGCgaqeaaaa@2E8B@_*S *_do not show monotonic variations as a function of *n*. At this point, one can argue that there is a significant role played by the precise structure used as target. This point will be made clearer when we analyze the time that a population needs for fixation of the target structure.

#### Search and fixation times

The statistically stationary state can be equated with an optimized state where the population is maximally functional, considering the restrictions imposed by the system parameters. From an evolutionary point of view, it is extremely important to quantify the time required to achieve the stationary state under different conditions. Adaptation to new environments is fully dependent on the ability of the population to respond in a time span comparable to that of the external fluctuations, and evolution apparently selects mutation rates that permit this long-term adaptation [23-25].

This is our motivation to carry out a detailed analysis of the transient time needed to obtain a continuous presence of the target structure in the population, starting from a random initial pool of sequences. As we have seen, the population is capable of finding solutions to the problem of maintaining a given secondary structure only for values of the mutation rate below threshold. Nevertheless, this might not be enough to guarantee survival of the population if there is not a persistent presence of a finite fraction of molecules folding into the target structure after a reasonably short number of generations.

Evolutionary success thus corresponds to appearance and fixation of the target structure in the population. We determine the time to success as the generation *g*_*F *_after which at least one molecule of the population folds into the target structure uninterruptedly for 500 generations. Even for low mutation rates, the dispersion of those values is large, and therefore we perform 200 independent runs to calculate *g*_*F*_.

**Figure 5:**
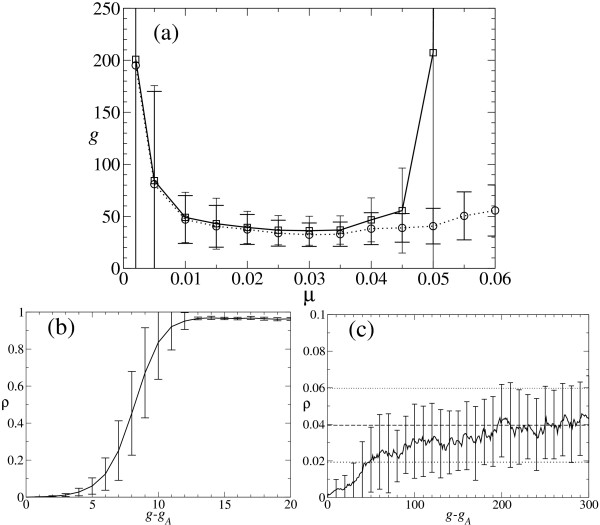
**Search and fixation time (in number of generations) as a function of *μ***. (a) Curves corresponding to average values of the search time *g*_*A *_when the target structure shows up for the first time in the population (dotted curve) and the total time *g*_*F *_of search plus fixation (solid curve). Most of the transient corresponds to searching time except for values of *μ *close to the threshold, where the limiting step is fixation. (b) Fixation time for *μ *= 0.002. After appearance (*g*_*A*_), it takes few generations to attain stationary values (ρ¯
 MathType@MTEF@5@5@+=feaafiart1ev1aaatCvAUfKttLearuWrP9MDH5MBPbIqV92AaeXatLxBI9gBaebbnrfifHhDYfgasaacH8akY=wiFfYdH8Gipec8Eeeu0xXdbba9frFj0=OqFfea0dXdd9vqai=hGuQ8kuc9pgc9s8qqaq=dirpe0xb9q8qiLsFr0=vr0=vr0dc8meaabaqaciaacaGaaeqabaqabeGadaaakeaaiiGacuWFbpGCgaqeaaaa@2E8B@ = 0.965 ± 0.008). (c) Fixation time for *μ *= 0.05 (ρ¯
 MathType@MTEF@5@5@+=feaafiart1ev1aaatCvAUfKttLearuWrP9MDH5MBPbIqV92AaeXatLxBI9gBaebbnrfifHhDYfgasaacH8akY=wiFfYdH8Gipec8Eeeu0xXdbba9frFj0=OqFfea0dXdd9vqai=hGuQ8kuc9pgc9s8qqaq=dirpe0xb9q8qiLsFr0=vr0=vr0dc8meaabaqaciaacaGaaeqabaqabeGadaaakeaaiiGacuWFbpGCgaqeaaaa@2E8B@ = 0.039 ± 0.020, shown as dashed and dotted horizontal lines). The probability to lose the target structure after its first appearance is high, fixation becomes difficult and, even if the asymptotic regime is reached on average, population fluctuations can lead to the occasional disappearance of the target structure. This is quantified by the very large fluctuations displayed by *ρ*, here shown as error bars. Simulations have been made for the hairpin structure, *N *= 602, and averages of 200 (a) and 25 (b, c) realizations have been performed.

In Fig. 5(a), we show the curves corresponding to the number of the generations *g*_*F *_to success as defined above and to the generation *g*_*A *_where a sequence folding into the target structure shows up for the first time. In principle, it appears equally difficult to maintain the target structure in the population at too low values of *μ *or at values close to the error threshold *μ*_*c*_. However, there are two rivaling processes, diversity generation and competition between mutants, which dominate the behavior of *g*_*F *_at each extreme (and are responsible for the asymmetric behavior of *g*_*A *_at low and high values of *μ*). For low values of *μ*, mutants appear rarely. In this situation, any mutation decreasing the distance to the target is certainly fixed in the population, since the copies of the advantageous mutant retain the advantage of its progenitor. For *μ *→ *μ*_*c*_, on the contrary, new mutants appear continuously. However, the advantages they might occasionally acquire are often wiped out, since their sequences are not maintained for long in the progeny. This slows down the spread of adaptive changes, which are then fixed with difficulty, to the whole population.

Figures 5(b) and 5(c) illustrate how fixation proceeds for low and high *μ*. For the scope of these figures, we have rescaled time and set as generation number 1 that at which the target structure appears for the first time in the population (*g*_*A*_). Then, for each generation number, we have averaged the density *ρ *over 25 realizations. We will discuss when fixation occurs and when the asymptotic regime is reached.

As can be seen in Fig. 5(b), the limiting factor at low *μ *is the searching time, as already discussed. Once the target structure is found, the number of correct sequences grows first exponentially, then saturates, and the stationary value of *ρ *is attained in a few generations. Specifically, for *μ *= 0.002 the correct structure appears first after 195 generations, is fixed 6 generations later, and the asymptotic regime is reached approximately after another 6 generations, i.e. after 207 generations (these are average values).

This is in contrast to the dynamics represented in Fig. 5(c), for *μ *= 0.05. Since mutants appear at a high rate, the first appearance of the target structure takes only about 41 generations on the average. Actually, independent numerical studies corroborate that searching time attains minimum values at the error threshold [26]. However, fixation of the particular sequence with the proper structure becomes a hard task too close to the threshold. Disappearance of that and related sequences folding into the target structures is likely, due to the high mutation rate, and about 200 generations (207 for the sample studied) elapse on average before the structure is actually established in the population. The asymptotic regime is reached approximately 200 generations after the first appearance of the target structure, i.e. after 241 generations for this sample. Nonetheless, there is always a finite probability that the target structure is lost due to population fluctuations.

The two processes (search and fixation) are simultaneously optimized in a broad range of mutation rates where the values for *g*_*F *_are relatively low and do not vary strongly with *μ*. According to our results, those values are relatively distant from the error threshold, in the range of 30% to 50% of *μ*_*c *_in the studied examples.

**Figure 6:**
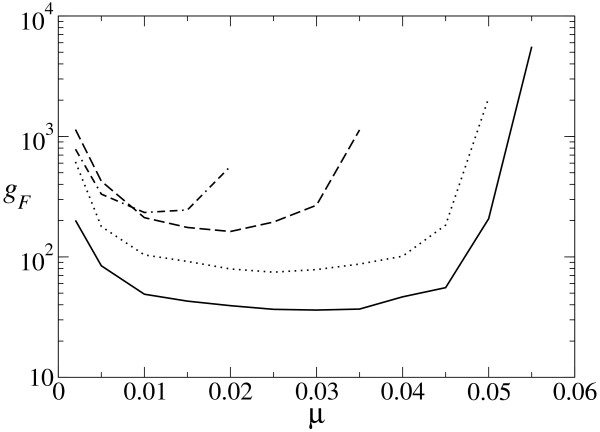
**Search and fixation time as a function of *μ *for different target structures**. The number of generations required to find and fix the target structure depends not only on the length of the sequence, but also on its specific secondary structure. It takes longer to fix a hammerhead structure than a hairpin structure of the same length. The interval of *μ *values where the structure can be effectively fixed shrinks as *n *increases. Simulations have been made for the hairpin structure (solid line), hammerhead structure (dotted line), 3-stem-loop structure (dashed line), model tRNA structure (dotted-dashed line), *N *= 602, and averages of 200 realizations have been performed.

We also have studied the behavior of *g*_*F *_with respect to the secondary structure chosen as target. As we show in Fig. 6, *g*_*F *_diverges for *μ *→ 0, *μ *→ *μ*_*c*_, and presents a minimum at intermediate values of *μ *for all investigated target structures. We have already seen above that *μ*_*c *_depends on *n *and on the specific structure studied.

Simulations not displayed here show that the qualitative behavior of *g*_*F *_does not depend on the system size *N*. However, some interesting quantitative differences deserve discussion. First, the time of first appearance *g*_*A *_shortens as *N *increases due to the higher diversity generated: a larger population undergoes more extensive searches in sequence space. On the other hand, fixation time remains essentially unchanged, since for most values of *μ *advantageous mutations spread exponentially fast. As a result, *g*_*F *_also decreases as *N *increases. This is in agreement with recent analytical results on the expected fixation time in asexual populations where clonal interference dominates adaptation. For large populations, and assuming that all mutations have the same effect on fitness, it can be shown that adaptation speed depends logarithmically on the population size [27].

A side effect of considering large populations evolving towards short molecules, as the ones here used, is that there is a non negligible probability that, solely by chance, one of the sequences folds into the target structure, an effect enhanced through the action of selection. This implies that the curve of *g*_*F *_broadens with *N*, as observed. However, for values of *μ *above *μ*_*c *_this is a spurious effect that is not accompanied by true fixation.

#### Structural robustness

**Figure 7:**
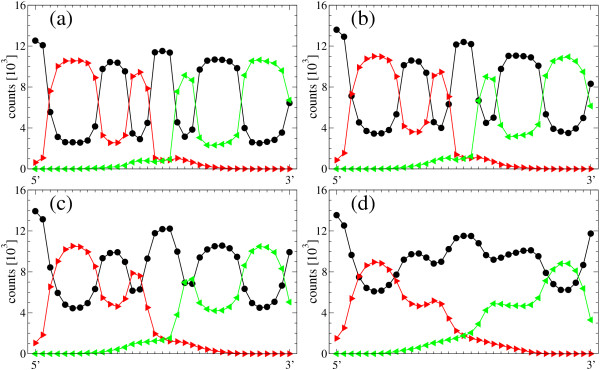
**Structural stability: disintegration of collective information depends on the secondary structure, example hairpin**. We represent, for each position along the sequences in the population, the total number of secondary structures presenting each structural state. In this case, we have only considered those sequences that do not fold into the hairpin structure. Subplots correspond to increasing values of the mutation rate *μ *= 0.04 (a), *μ *= 0.05 (b), *μ *= 0.06 (c), and *μ *= 0.07 (d). This representation permits to identify robust motifs in the secondary structure, that is, structural parts that are maintained at high values of *μ*. Curves have been calculated from the asymptotic state (after 6000 generations) of 25 simulations for a population with *N *= 602. From the obtained 15050 structures, those folding into the target structure have been discounted: 1891 (a), 590 (b), 70 (c), 0 (d). Black dots denote unpaired positions ".", red triangles directed to the right correspond to upstream pairs "(", and green triangles directed to the left are downstream paired ")" nucleotides. The consensus structure is obtained by taking the most frequent state at each position.

We have described the process of loss of phenotypic information when the mutation rate approaches its critical value. Close to the error threshold, the fraction of sequences correctly folding into the target secondary structure is low, while the density of the consensus structure is essentially one. Remarkably, this remains true even if all correctly folded sequences are not included in the analysis. Results on the structural robustness of the evolutionary process are summarized in Figures 7 (hairpin) and 8 (hammerhead).

**Figure 8:**
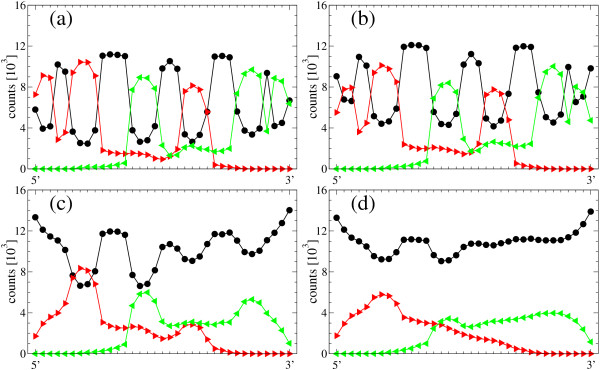
**Structural stability: disintegration of collective information depends on the secondary structure, example hammerhead**. The same as for Fig. 7, but for the hammerhead structure and mutation rates *μ *= 0.04 (a), *μ *= 0.05 (b), *μ *= 0.06 (c), and *μ *= 0.07 (d). Correctly folding sequences have been discounted: 1987 (a), 484 (b) and 0 (c, d).

We analyze the structure of the population after the asymptotic regime has been reached. To this aim, we represent, for each position along the sequences in the population, the total number of secondary structure states, i.e. unpaired, paired upstream and paired downstream. The diagrams are obtained for four different values of *μ *(below and above *μ*_*c*_) and based on 25 realizations each. We only consider those sequences that do not fold into the target structure, in order to emphasize the presence of a distributed, collective but delocalized state of the population that retains information on the selective pressure to which it is subjected.

For example, for the hairpin structure and *μ *= 0.04 (Fig. 7(a)), we find at the 5' position 12532 unpaired, 627 upstream paired and 0 downstream paired structural states. This representation is analogous to that obtained from sequencing analyses, since the consensus structure corresponds to the most frequent state at each position. Through averaging, we can recover a virtual hairpin structure even from the sequences that do not fold into hairpin structures (as it occurs, e.g. in Fig. 7(a)).

Despite the fact that above the error threshold the target structure is practically not present within the population, parts of the structure are recognized in population averages. It is remarkable that the structure does not disappear at once: certain structural elements are weaker and disappear at lower mutation rates, but others are maintained. Our detailed studies with the hairpin and the hammerhead structures show that long stacks are amongst the most stable elements, while short stacks linking loops disappear more easily (see Figs. 7(d) and 8(c)).

#### Selective pressure

All numerical results presented in this study have been obtained for *β *= 1. However, we also have performed simulations for different values of the selective pressure *β*. The larger *β*, the easier it is to fix the target structure in the population, and the larger are the asymptotic values of ρ¯
 MathType@MTEF@5@5@+=feaafiart1ev1aaatCvAUfKttLearuWrP9MDH5MBPbIqV92AaeXatLxBI9gBaebbnrfifHhDYfgasaacH8akY=wiFfYdH8Gipec8Eeeu0xXdbba9frFj0=OqFfea0dXdd9vqai=hGuQ8kuc9pgc9s8qqaq=dirpe0xb9q8qiLsFr0=vr0=vr0dc8meaabaqaciaacaGaaeqabaqabeGadaaakeaaiiGacuWFbpGCgaqeaaaa@2E8B@ for the same mutation rate. Nevertheless, the results do not qualitatively depend on the specific value of *β *as long as it takes values around unity. The process changes qualitatively in either of the limits *β *→ 0 and *β *→ ∞. In the first case, selection does not discriminate between structures closer or further from the target structure, and the dynamics become a random branching process for the original set of sequences. Since no advantage is given to any particular sequence, it is not possible to fix sequences close to the target and the phenotypic error threshold *μ*_*c *_→ 0. In the second case, an infinitely large value of the selection pressure amounts to assigning a probability of replication 1 to the sequence closest to the target and zero to any other sequence. Fixation proceeds fast, but searching relies strongly on random events, and thus becomes a lengthy task. Some analytical results have been derived in this limit [28].

If we use the Hamming distance instead of the base-pair distance, search and fixation times become significantly larger. This reflects the fact that the base-pair distance penalizes more strongly structures far from the target than does the Hamming distance. According to the latter, two structures can be apparently close but still at a large evolutionary distance, a fact that is better captured by the base-pair distance. The effect of a worse distance measure can be partly compensated by increasing *β*, as Eq. (1) reveals: the two parameters have indeed an interchangeable effect on selection. From a phenomenological point of view, either *d *or *β *take implicitly into account the selective pressure of the environment, where different mechanisms can be at work to discriminate between optimally and sub-optimally performing structures. Finally, let us emphasize that a given functional form of the selective pressure acting on any evolutionary system of this kind has a curve ρ¯
 MathType@MTEF@5@5@+=feaafiart1ev1aaatCvAUfKttLearuWrP9MDH5MBPbIqV92AaeXatLxBI9gBaebbnrfifHhDYfgasaacH8akY=wiFfYdH8Gipec8Eeeu0xXdbba9frFj0=OqFfea0dXdd9vqai=hGuQ8kuc9pgc9s8qqaq=dirpe0xb9q8qiLsFr0=vr0=vr0dc8meaabaqaciaacaGaaeqabaqabeGadaaakeaaiiGacuWFbpGCgaqeaaaa@2E8B@(*μ*) associated. As long as different environments mean different forms and strengths for the selective pressure, they will define different values for *μ*_*c*_. That is to say, the value of the error threshold is a function not only of the sequence length, but also of the precise environment [29].

### Comparison with experimental observations in molecular quasispecies

#### Sequence heterogeneity

The theoretical framework considered here can be compared with the experimental knowledge derived from the study of molecular or viral quasispecies. Natural RNA populations harbor large degrees of diversity regarding sequence composition, as it was already documented in the first systematic genetic analyses performed on viral quasispecies. They showed that, in multiply passaged populations of Q*β *phage, each viable virus differs in one or two positions (out of less than 4500 nucleotides in the whole genome) from the consensus sequence [14]. The large heterogeneity of real quasispecies at the genetic level was confirmed by further studies, which established that RNA viruses are ensembles of mutants resulting form error-prone replication processes with average mutation rates in the range of 10^-3 ^to 10^-5 ^base substitutions per nucleotide copied [30]. Nevertheless, such a high mutation rate does not necessarily place the quasispecies at the very edge of the error threshold, as often assumed. Experiments using chemical mutagens have demonstrated that RNA viruses can increase their mutation rates up to 2.8-fold in riboviruses [15] and up to 13-fold in retroviruses [31,32] without compromising their viability. Increases in the mutation rate of quasispecies as a natural strategy to optimize the exploration of the sequence space have been also documented through the description of hypermutated (though still viable) viral genomes in *in vivo *infections (reviewed in [33]).

Regarding molecular populations derived from directed RNA evolution *in vitro*, most of the experiments so far performed have focused on the evolutionary outcome (the selected ribozyme or aptamer) rather than on the process itself (reviewed in [34] and [35]). However, some of the experimental approaches have included the analysis of clonal sequences derived from different transfers of continuous evolution or rounds of stepwise evolution. In those cases, it was possible to track the evolution of RNA genotypes (by means of the comparative analysis of each isolated sequence) and/or phenotypes (by analyzing their corresponding secondary structure or catalytic/binding activity) over time. Following this combined approach, the first mutation-accumulation experiment with *in vitro *evolving populations of RNA molecules was recently performed [36]. In parallel, comparative analysis of real ribozymes subject to extensive mutagenesis have shown that the mutation error rate actually selected during RNA evolution is well below threshold, since structural and functional considerations put it up to eight times larger [37] than the error expected from classical quasispecies theory, where separation between genotypic and phenotypic levels is not taken into account [38].

Moreover, when genotype and phenotype are decoupled in models of evolution, it turns out that the most abundant sequence does not necessarily coincide with the master sequence, i.e. the fittest sequence, as defined in quasispecies theory. This agrees with experimental observations demonstrating that even if a population accumulates mutations steadily through bottleneck events, its fitness can achieve high values comparable to those of an optimized initial population [16,39]. That is to say, there are many different genotypes which yield comparable levels of biological performance.

#### Secondary structures

In parallel to the genotypic information derived from experimental work on quasispecies, a large effort has also been devoted to their structural analysis. Besides RNA secondary structure prediction using folding algorithms [18,40] or comparative sequence analyses [41], several experimental techniques have been developed to test *in vitro *the actual secondary structure of RNA molecules from homogeneous (such as cellular ribosomal RNA) or heterogeneous (including fragments of genomic viral RNA) populations. Two of the most widespread experimental techniques for secondary structure determination in viral RNA involve either the use of RNases with different specificity or the chemical modification of RNA followed by primer extension. As an example, both approaches have been successfully applied to the analysis of the very structurally compact internal ribosome entry sites (IRES) elements in viral RNA, and they have allowed the characterization of functionally relevant tertiary interactions [42,43]. Recently, an alternative method of RNA structure analysis has been developed, based on the hybridization of RNA in native conditions to microarrays of complementary DNA oligonucleotides [44,45]. Additionally, the three-dimensional solution structure of different biologically active RNAs can be experimentally determined by means of different approaches, including X-Ray diffraction techniques [46] and Nuclear Magnetic Resonance-based methods [47].

In the case of heterogeneous RNA populations such as molecular or viral quasispecies, the consensus sequence can be easily obtained by means of population sequencing, and the corresponding secondary structure of the region under study can be predicted [48,49]. The experimental determination of the structure corresponding to the consensus sequence using the above mentioned methodologies would require the (chemical or enzymatic) synthesis of a real homogeneous population of RNA molecules harboring a sequence identical to the consensus of the population. Therefore, in general, folding algorithms are used for the prediction of the structure corresponding to the consensus sequence, whereas experimental structural techniques can be applied to the determination of the structure corresponding to the most abundant sequence in the population. In both cases, functional and evolutionary consequences can be derived from that structural information. In our simulations, we have observed that the consensus sequence folds into the target structure most of the times. However, this does not imply that individual molecules in the population do it with the same probability. On the contrary, high values of ρ¯
 MathType@MTEF@5@5@+=feaafiart1ev1aaatCvAUfKttLearuWrP9MDH5MBPbIqV92AaeXatLxBI9gBaebbnrfifHhDYfgasaacH8akY=wiFfYdH8Gipec8Eeeu0xXdbba9frFj0=OqFfea0dXdd9vqai=hGuQ8kuc9pgc9s8qqaq=dirpe0xb9q8qiLsFr0=vr0=vr0dc8meaabaqaciaacaGaaeqabaqabeGadaaakeaaiiGacuWFbpGCgaqeaaaa@2E8B@_*C *_can be obtained for relatively high mutation rates, where very few sequences in the population are functional.

If one is interested in the functionality of a particular RNA sequence within the mutant spectrum of the quasispecies, it can be picked-up from the population following a protocol based on molecular cloning and sequencing, that allows the sequence analysis of both majority and minority genomes of the quasispecies, as recently exemplified with human immunodeficiency virus [50]. The secondary structure corresponding to each of the sequences can be determined as described above, with an experimental limitation imposed by the number of molecular clones that can be obtained and analyzed. This limitation could be circumvented in the future by means of DNA microarray technology optimized to allow the quick characterization of sequences (and, eventually, structures) of minority genomes within the quasispecies. Therefore, depending on the completeness of the analysis performed, these approaches could lead to the experimental determination of the consensus structure of natural populations.

## Conclusion

The mutation rate characteristic of a natural quasispecies has been selected through evolutionary optimization. The observed value probably minimizes adaptation time, which has to combine two opposing trends: one tries to increase the mutation rate in order to generate diversity and the other pushes to decrease the mutation rate to fix readily and maintain fitter variants in the population. The exploration of the sequence space in search of improved phenotypes is a collective process. As such, some properties of the quasispecies cannot be ascribed to a particular sequence, not even to the most abundant one, but are revealed only when averages over the population are performed. In its collective adaptation towards a target structure, the quasispecies keeps a distributed and delocalized global state that informs about the selective pressures driving the evolutionary process. Such structural robustness is also recognizable through the progressive loss of phenotypic information above the error threshold.

Our observations might be relevant when considering early stages of molecular evolution in the context of an RNA world, where high mutation rates at replication could not be avoided. As a result, the shorter the sequences of a population, the more probable that they could stably maintain their functionality. Stability would improve as well in the presence of large populations and of an effective selection mechanism.

Remarkably, certain structures would also favour fixation at high *μ*. As we have seen, function encoded in hairpin-like structures could be selected in longer sequences, for the same mutation rate, or at higher mutation rates, for fixed molecular length. The hairpin motif is frequent in nature [51], apart from being one of the most common structures, attending to the probability that it results from folding of a random sequence. We conjecture that certain secondary structure motifs, if functional, could be frozen accidents in evolution, in the sense that they were much more prone to appear in short molecules subjected to high mutation rates, and thus became fixed and subsequently used as building blocks of more complex molecules [20].

## Methods

Simulations have been carried out at the Itanium II cluster of INTA (Instituto Nacional de Técnica Aeroespacial, Spain). For random number generation, we relied on the Mersenne Twister and Ziff's GFSR4 algorithms as provided by GNU Scientific Library (GSL), Version 1.7 [52]. For secondary structure folding (minimum free energy) and calculation of base-pair and Hamming distances, we use the Vienna RNA package [18], version 1.5, with the current standard parameter set.

## Authors' contributions

All authors conceived and designed the research, analyzed the data and wrote the paper. MS performed the simulations. All authors read and approved the final manuscript.
